# A pilot study on the effect of SARS-CoV-2 spike protein on IL-1β-mediated inflammation in peripheral blood immune cells from AIED patients

**DOI:** 10.1186/s10020-025-01227-0

**Published:** 2025-05-06

**Authors:** Shresh Pathak, Natalie Tan, Andrea Vambutas

**Affiliations:** 1 Northwell Health, 2000 Marcus Avenue, New Hyde Park, New York, NY 11042 USA; 2 Feinstein Institutes for Medical Research, Manhasset, New York, NY USA; 3 Department of Otolaryngology, Donald and Barbara Zucker, School of Medicine, Hofstra/Northwell, Hempstead, New York, NY USA; 4 Department of Molecular Medicine, Donald and Barbara Zucker School of Medicine at Hofstra/Northwell, Hempstead, 350 Community Drive, Manhasset, New York, NY 11030 USA; 5 Albert Einstein College of Medicine, Bronx, New York, NY USA

## Abstract

**Background:**

Immune-mediated hearing loss (IMHL) patients (comprised of autoimmune inner ear disease (AIED) and sudden sensorineural hearing loss (SSNHL)) may be at higher risk for hearing loss following Coronavirus disease (COVID-19) infection and/or vaccination.

**Methods:**

We compared inflammatory cytokine expression in response to SARS-CoV2 spike protein between two groups of patients with IMHL: IMHL patients that temporally demonstrated worsening SNHL following COVID vaccination or infection as compared to IMHL patients with worsening SNHL unrelated to COVID exposure: (IMHL-COVID ( +)) (*n* = 11) (IMHL-COVID (-)) (*n* = 10). In these two groups, we treated isolated PBMCs with increasing amounts of SARS-CoV-2 spike protein and compared responses to stimulation with positive and negative controls.

**Results:**

Peripheral Blood Mononuclear Cells (PBMC) from IMHL-COVID ( +) patients had increased expression and release of both IL-1β and IL-6 in response to spike protein as compared to IMHL-COVID (-) patients. However, when the IMHL-COVID ( +) group was broken down into AIED patients compared to SSNHL, it became apparent that the greatest responses were from the AIED patients (*p* < 0.005 for IL-6 mRNA expression and *p* < 0.003 for IL-6 release when compared between any two similar groups using Wilcoxon Rank-Sum Test).

When we broke down the COVID ( +) group to infection versus vaccination, the immune responses in the infection group (*N* = 3 AIED, 1 SSNHL) were stronger.

**Conclusions:**

COVID-19 exposure with reported changes in hearing sensitivity in IMHL patients resulted in pro-inflammatory responses in response to spike protein. The inflammatory responses were greatest in AIED patients, and greater following infection rather than vaccination. Therefore, based on these studies, we would recommend AIED patients take additional precautions to avoid COVID exposure. Furthermore, we do recommend COVID vaccination during periods of hearing stability, as the immune responses are even more robust in response to infection in this vulnerable group.

## Background

SARS-CoV-2 infection was declared a pandemic around 5 years ago by the World Health Organization (WHO). COVID-19 infection has exerted extensive effects on virtually all organs Yao 2021; Sood and Bedi 2022; Shah 2021, including the cochlea Jeong 2021. Otolaryngologists have reported increased numbers of patients reporting changes in auditory acuity following COVID-19 infection Ríos Coronado 2023; Fancello 2022 or COVID-19 vaccination Colizza 2022. Reports linking COVID-19 infection to sudden hearing loss have emerged suggesting increased susceptibility for SSNHL in patients with COVID-19 Chern et al. 2021; –Degen et al. 2020. Two recent studies explored the risk of sudden sensorineural hearing loss (SSNHL) post COVID-19 vaccination with conflicting results. In one study a positive correlation was identified, where the association between the BNT162b2 mRNA COVID-19 vaccine might be linked with increased risk of SSNHL although the effect size was very small Yanir et al. 2022. Conversely, Formeister et al*.*did not identify a correlation between COVID-19 vaccination and an increased frequency of sudden hearing loss compared with the known incidence in the general population Formeister 2022. Similarly, a national registry retrospective cohort study of 5.5 million Finnish residents did not show any increased risk for SSNHL after any COVID-19 vaccination Nieminen 2023. Similarly in a recent study despite of employing a randomized sample, the results did not support the linkage of COVID-19 illness or vaccination to sudden sensorineural hearing loss Thompson et al. 2024. Given reports linking both infection and vaccination to the potential development of sensorineural hearing loss, the potential causative agent likely is the expression of the SARS CoV2 spike protein, as all the current vaccines target the spike protein. SARS-CoV-2 enters the cell through angiotensin-converting enzyme 2** (**ACE2) receptor Violi et al. 2020 permitting infection of the central nervous system, the peripheral nervous system, and auditory cortex of the temporal lobe Mao 2020. Human inner ear tissues have been demonstrated to express the ACE2 receptor, as well as the transmembrane protease serine (TMPRSS) and FES upstream region (FURIN) required for cellular entry Jeong 2021.

Patients with AIED have multiple periods of sensorineural hearing decline triggered by yet unknown stimuli unlike patients with SSNHL where the patient experiences a single acute decline in hearing. The correlation between hyperactive immune system due to COVID-19 and the generation of autoimmunity has been demonstrated Mohandas 2023. Patients with autoimmune disease typically are hyper-responsive to many environmental stimuli Zhao 2019; –Pathak et al. 2013, and therefore, potentially at greater risk from COVID-19 exposure. In support of this, in a study done by Wichova et al. that demonstrated sudden hearing loss following COVID-19 exposure, approximately one third of their 30 patients had previous otologic diagnoses such as autoimmune inner ear disease (AIED) or Meniere’s disease (MD) Wichova et al. 2021. New onset autoimmunity has been reported following COVID-19 mRNA vaccination Chen 2022, and therefore, mRNA vaccines may act as either an antigen or adjuvant through nucleotide-binding domain, leucine-rich–containing family, pyrin domain–containing-3 (NLRP3), which is a sensing molecule that is part of the interleukin-1 cascade Kelley et al. 2019. A recent study using apoptosis-associated speck-like protein containing a caspase-associated recruitment domain (CARD) Asc^−/−^ and Nlrp3^−/−^mice showed no substantial change in antibody or T cell responses in comparison to wildtype mice indicating that immunogenicity of BTN162b2 (a lipid nanoparticle-formulated, nucleoside-modified RNA vaccine that encodes a prefusion stabilized, membrane-anchored SARS-CoV-2 full-length spike protein) could be mediated by inflammasome independent pathway Li 2022.

In the present study, we investigated the inflammatory responses in patients describing an exacerbation of sensorineural hearing loss in IMHL patients that occurred within a 2-week period following COVID-19 infection and COVID-19 vaccination Formeister 2022. Given our previous findings of elevated levels of interleukin-1β (IL-1β) Pathak et al. 2011 and interleukin-6 (IL-6) Gorthey et al. 2021 in corticosteroid-unresponsive AIED patients, we have chosen to use cytokine response in PBMCs as a marker of immune activation in this study. We also queried whether the IL-1 receptor antagonist (IL-1Ra) was modulating the inflammatory response in these patients as it serves to regulate inflammation. We compared IL-1β, IL-1Ra and IL-6 mRNA production and release between IMHL patients with worsening SNHL after COVID-19 vaccination or infection (COVID (+)) with IMHL patients with worsening SNHL unrelated to COVID-19 (COVID (-)). To begin to establish a causal role between SARS-CoV-2 spike protein and the pro-inflammatory cytokines IL-1β and IL-6 we studied expression of these molecules by stimulating peripheral blood mononuclear cells (PBMCs) with increasing dose of SARS-CoV-2 spike protein and compared responses to stimulation with myelin basic protein (MBP), and lipopolysaccharide (LPS) samples. MBP was used as a non-specific antigen in our studies which is an antigen in Multiple Sclerosis (MS), another autoimmune disease with neurologic manifestations Hedegaard 2009.

## Materials and methods

### Patient sample selection and ethical statement

#### Ethics approval and informed consent

The procedures using human biological samples were performed in accordance with institution regulations of Northwell Health System Institutional Review Board (IRB). All procedures were performed in accordance with ethical guidelines. Written informed consent was provided by all enrolled patients prior to entering the study. Patients were enrolled at the time of their office visit reporting a decline in hearing, and blood was collected if audiometric confirmation of an asymmetric hearing loss was obtained. All patients were queried as to COVID-19 exposure, COVID-19 vaccination history, and hearing health history of IMHL which included prior diagnoses of SSNHL, AIED or Meniere’s Disease. The hearing losses all occurred between 2021 and 2023. In all AIED patients, prior audiograms were available to confirm the acute change in hearing that was temporally related to COVID-19 exposure. In SSNHL patients, the presence of asymmetric sensorineural hearing loss was used as criteria for inclusion as prior audiometric data was not available. Patients were excluded if they had MRI findings of retrocochlear pathology or other etiologies of sensorineural hearing loss.

Of the twenty-one patients enrolled in these studies, all were Caucasian. The IMHL-COVID (-) cohort of patients were comprised of 10 patients, ranging in age from 32–69 years of age, with an equal number of males and females. The clinical corticosteroid response rate was 40% representing the rate of hearing improvement following the administration of steroids. The IMHL-COVID (+) cohort of patients was comprised of eleven subjects ranging in age from 22–72 years of age, with 6 males and 5 females. The clinical corticosteroid response rate was 27% for this cohort.

All patients had PBMC harvested immediately prior to the institution of corticosteroid therapy. The time interval between the index event of perceived loss and clinical evaluation and enrollment was highly variable, from 1 day to several months, however the perceived hearing change had to have occurred within a two-week period following infection/vaccination to be considered for enrollment. Informed consent was provided by all enrolled patients prior to entering a subject into the study. This study enrolled 21 IMHL patients out of which 11 reported a history of acute sensorineural hearing decline within a two-week period following COVID-19 vaccination or COVID-19 exposure. The IMHL-COVID (+) group is further broken down into 6 patients with AIED that experienced prior hearing declines over a several year period prior to COVID-19 exposure and 5 patients with SSNHL temporally related to COVID-19 exposure with no prior history of acute hearing loss. The demographics of the enrolled patients are shown in Table 1. The 10 COVID (-) patients all had AIED with prior hearing declines prior to the COVID-19 pandemic as demonstrated by audiometric changes (variable dates) and had previously undergone uneventful vaccination for COVID-19. Audiometric patterns, and corticosteroid response is described. For the COVID (-) group, blood was collected from patients with an acute decline in hearing, however only if they had at least 1 prior COVID vaccination that was uneventful.

**Table 1:** Patient characteristics, audiometric patterns in patients with immune mediated hearing loss

Patient	Age	RACE/ GENDER	Diagnosis	Hearing loss post vaccination or infection	Hearing loss	Loss related to COVID?/COVID VACCINE?	Previous hearing declines unrelated to covid?	Hearing Recovery with steroids?
S01	69	WM	MD	NO	ASYMMETRIC UPSLOPING	NO	YES	YES
S02	58	WF	AIED	NO	ASYMMETRIC RECENT FLAT, DOWNSLOPING	NO	YES	NO
S03	43	WF	AIED	NO	ASYMMETRIC UPSLOPING	NO	YES	NO (Y W/ANAKINRA)
S04	32	WM	MD	NO	UNILATERAL UPSLOPING	NO	YES	NO
S05	54	WM	AIED	NO	ASYMMETRIC	NO	YES	YES
S06	65	WF	AIED	NO	ASYMMETRIC UPSLOPING	NO	YES	NO
S07	62	WM	AIED	NO	UPSLOPING	NO	YES	YES
S08	63	WF	AIED	NO	FLAT SYMMETRIC	NO	YES	NO
S09	64	WM	AIED	NO	ASYMMETRIC	NO	YES	NO
S10	65	WF	AIED/MD	NO	ASYMMETRIC FLAT	NO	YES	YES
S11	50	WM	SSNHL	YES, VACCINATION, 2 DAYS POST J & J	HF SYMMETRIC SNHL	YES	NO	NO
S12	60	WM	AIED	YES, VACCINATION, 2 WEEKS POSTJ &J	ASYMMETRIC FLAT	YES	YES	NO
S13	73	WM	AIED	YES, INFECTION, WITHIN 2 WEEKS POST COVID INFECTION	ASYMMETRIC DOWNSLOPING	YES	YES	NO
S14	52	WF	SSNHL	YES, INFECTION, POST COVID INFECTION (TESTED POSITIVE 2 DAYS AFTER HEARING LOSS)	DOWNSLOPING	YES	NO	YES
S15	72	WF	SSNHL	YES, VACCINATION, IMMED FOLLOWING 2 ND PFIZER VACCINE	ASYMMETRIC FLAT	YES	NO	NO
S16	22	WM	AIED	YES, INFECTION, COINCIDENT WITH COVID INFECTION	DOWNSLOPING	YES	YES	NO
S17	51	WF	AIED	YES, VACCINATION, 1 WEEK POST MODERNA BOOSTER	ASYMMETRIC FLAT	YES	YES	NO
S18	40	WF	AIED	YES, INFECTION, FOLLOWING FIRST PFIZER VACCINE & LATER DEVELOPED COVID WITH 2 ND DECLINE	ASYMMETRIC UPSLOPING	YES	YES	NO/NO
S19	61	WF	SSNHL	YES, VACCINATION, 3 DAYS POST 4 TH MODERNA BOOSTER	UNILATERAL FLAT	YES	NO	NO
S20	71	WM	SSNHL	YES, VACCINATION, 2 DAYS POST 2 ND PFIZER BOOSTER	DOWNSLOPING ASYMMETRIC	YES	NO	YES, BUT DELAYED
S21	48	WM	AIED	YES, VACCINATION, 2 WEEK POST PFIZER BOOSTER	ASYMMETRIC UPSLOPING	YES	YES	YES

### Experimental procedures

#### PBMC Isolation

Briefly, human PBMCs were isolated from buffy coats of IMHL patients using Ficol-paque Plus (Cytiva) density gradient centrifugation as described before Pathak et al. 2011. Cells were washed twice in RPMI 1640 medium, isolated cells were counted using a Z2 Coulter Particle Count and Size Analyzer (Beckman Coulter Life Sciences). PBMCs were cultured in RPMI 1640 medium with 4.1 mmol/L L-glutamine, 10% fetal bovine serum (FBS) (Atlanta Biologicals), and antibiotics (100 U/mL penicillin (Thermo Fisher Scientific) and 100 μg/mL streptomycin (Thermo Fisher Scientific) in the presence of 5% CO_2_ at 37 °C. While it may be impossible to identify and control all sources of variability in cell processing, such variability can impact cytokine measurement. Therefore, we used the same lot number of FBS, RPMI 1640 medium, and other reagents, and maintained the exact same incubation time throughout this study.

### Stimulation protocols-(stimulation with SARS-CoV2 spike protein and other treatments)

PBMCs were incubated at 1.0 × 10^6^ cells/mL for 16 h with SARS-CoV-2 spike protein (Sino biologicals) at the concentration of 12 μg/ml, 12 ng/ml and 12 pg/ml concentrations. 100 ng/ml LPS (Sigma), or 12 μg/ml myelin basic protein (MBP) (Biotechne). After incubation, the cells were collected and tested for viability by trypan blue exclusion using Cellometer (Nexcelom Bioscience). Samples were centrifuged and supernatants were collected and stored at − 70 °C.

Since the vaccine was released under an emergency use authorization use of the vaccines for research, even wasted vaccine, could not be obtained from either the vaccine manufacturers or from the New York State Department of Health for the current study therefore SARS-CoV2 spike protein was used for the current study.

### Methods for cytokine analysis

#### Quantitative-PCR (qPCR) for IL-1β, IL-6 and IL-1Ra mRNA expression

RNA was isolated from PBMCs of patient samples using RNAeasy Mini Kit (Qiagen) according to the manufacturer’s protocol. The qPCR was performed in a Roche Light Cycler 480 or ABI as previously described Pathak et al. 2011. The data was analyzed by the 2-ΔΔCT method. Primer pairs for IL-1β were designed using UPL Probe Library Assay Design Center. Primer sequences used for IL-1β were ctgtcctgcgtgttgaaaga (forward) and ttgggtaatttttgggatctaca (reverse). UPL probe number 78 was used with the primers. For Gene expression level of endogenous control GAPDH (Hs99999905_m1) and other transcripts (IL-6 (Hs00985639_m1), IL-1Ra (Hs00893626_m1)) were determined using pre-validated TaqMan Gene Expression Assays (Applied Biosystems).

### IL-1β, IL-6 and IL-1Ra levels in the culture supernatants by ELISA

The IL-1β, IL-6 and IL-1Ra levels in the culture supernatants were measured by sandwich ELISA as specified by the manufacturer (all from R&D Systems). The cells were exposed to different treatments and their controls as per requirements for 16 h in RMPI 1640 supplemented with 10% FBS in 24-well plates. The supernatants after brief centrifugation were then aspirated and stored at − 70 °C until assayed for IL-1β, IL-6 and IL-1Ra by ELISA. The concentration was determined using a 4-parameter logistic curve. At least two replicates were used for analysis for each experiment.

### Statistical analyses

Descriptive statistics, including the mean, standard deviation, median, interquartile range, minimum, and maximum, were calculated for continuous variables in the overall cohort and stratified by treatment. Similarly, frequencies and percentages were tabulated for categorical variables in the overall cohort and stratified by treatment. Comparisons between the two groups were performed using the Wilcoxon Rank-Sum Test for continuous variables. Due to the small sample size, the assumption of normality could not be reliably assessed, and a non-parametric test was deemed more appropriate. All analyses were carried out using SAS V9.4 (SAS Institute, Cary, NC). Given the exploratory nature of this study, a *p*-value < 0.01 was considered statistically significant to adjust for multiple comparisons.

## Results

### Patient recruited for studies

For this pilot study, twenty-one patients were recruited (Table 1). Ten patients with AIED that previously underwent uneventful COVID-19 vaccination were recruited during a period of active hearing decline (COVID (-). By comparison, 11 patients that reported hearing loss associated with COVID-19 exposure (COVID (+), were also recruited. The reported hearing loss temporally occurred no greater than two weeks following covid exposure (infection or vaccination), however the time to office presentation was variable. Five of these patients never experienced a prior hearing decline (SSNHL), whereas the other six patients had one or more prior hearing declines unrelated to COVID-19 exposure and were therefore classified as having AIED. The corticosteroid response rate for hearing recovery was lower in the IMHL-COVID (+) cohort (27 versus 40 percent). Notably, there was no uniform exposure to any one COVID-19 vaccine in this patient subgroup. Peripheral blood mononuclear cells (PBMC) were stimulated with either increasing amounts of spike protein and compared to LPS as a positive control or MBP as non-specific proinflammatory antigen.

### IL-6 is mildly expressed and robustly released in IMHL-COVID ( +) patients in response to spike protein

**Fig. 1:**
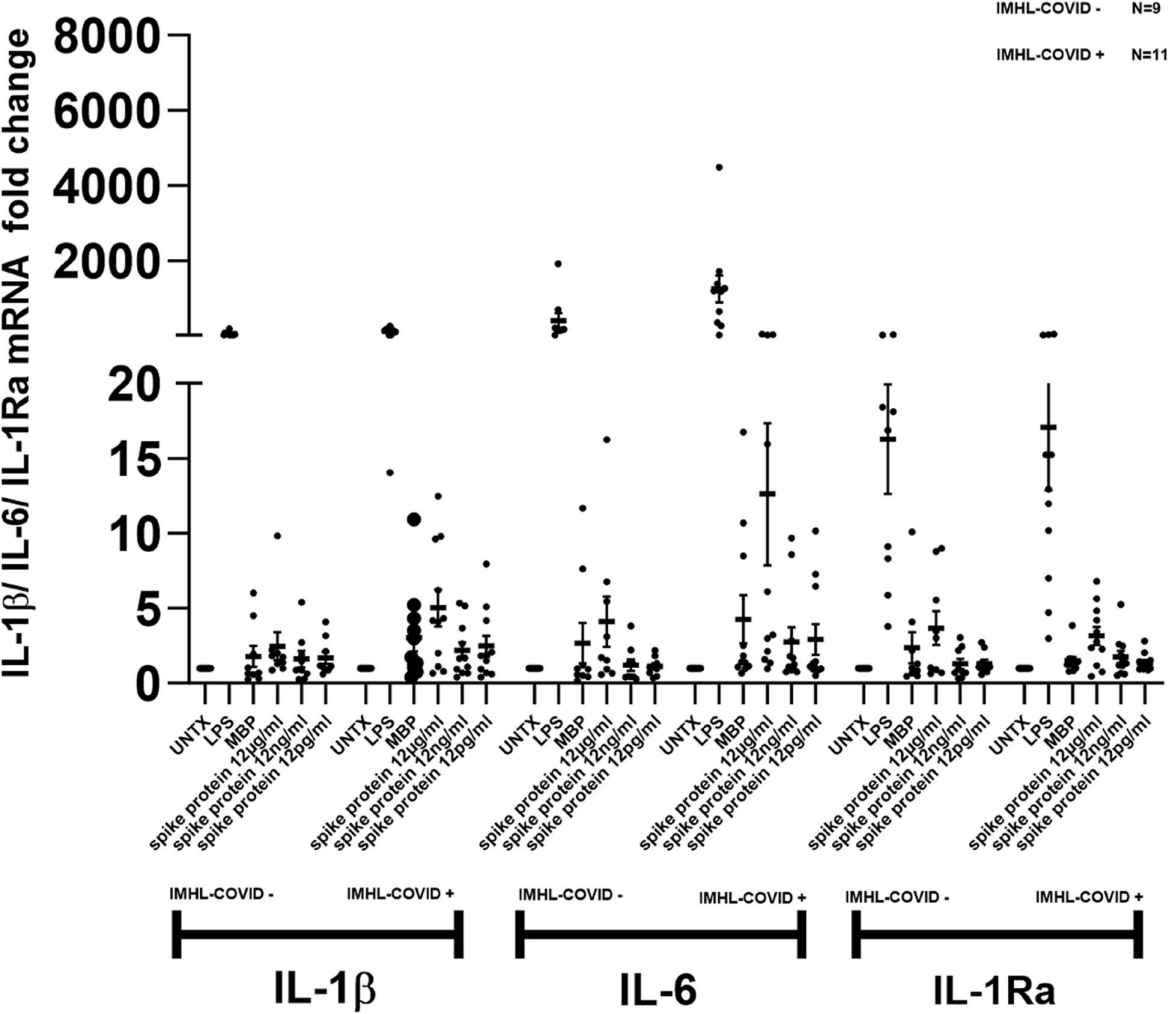
SARS-CoV-2 spike protein treatment resulted in stronger induction in IL-1β and IL-6 mRNA levels in the PBMCs of IMHL-COVID (+) group. PBMCs from IMHL-COVID (-) patients (*n* = 9) and IMHL-COVID (+) patients (*n* = 11) were treated with spike proteins at the concentrations of 12 μg/ml, 12 ng/ml, 12 pg/ml, MBP as nonspecific protein, LPS as positive control and compared with no treatment. The expression levels were analyzed by Q-RT–PCR. IL-1β—SARS-CoV-2 spike protein treatment resulted in mild increase in IL-1β mRNA levels in the PBMCs of both groups with slightly higher in IMHL-COVID (+) group. IL-6—SARS-CoV-2 spike protein treatment results in IL-6 mRNA induction in both groups and was more robust in IMHL-COVID (+) patients compared to IMHL-COVID (-) patients. IL-1Ra—SARS-CoV-2 spike protein treatment results in mild induction in IL-1Ra mRNA levels in the PBMCs of both groups

**Fig. 2:**
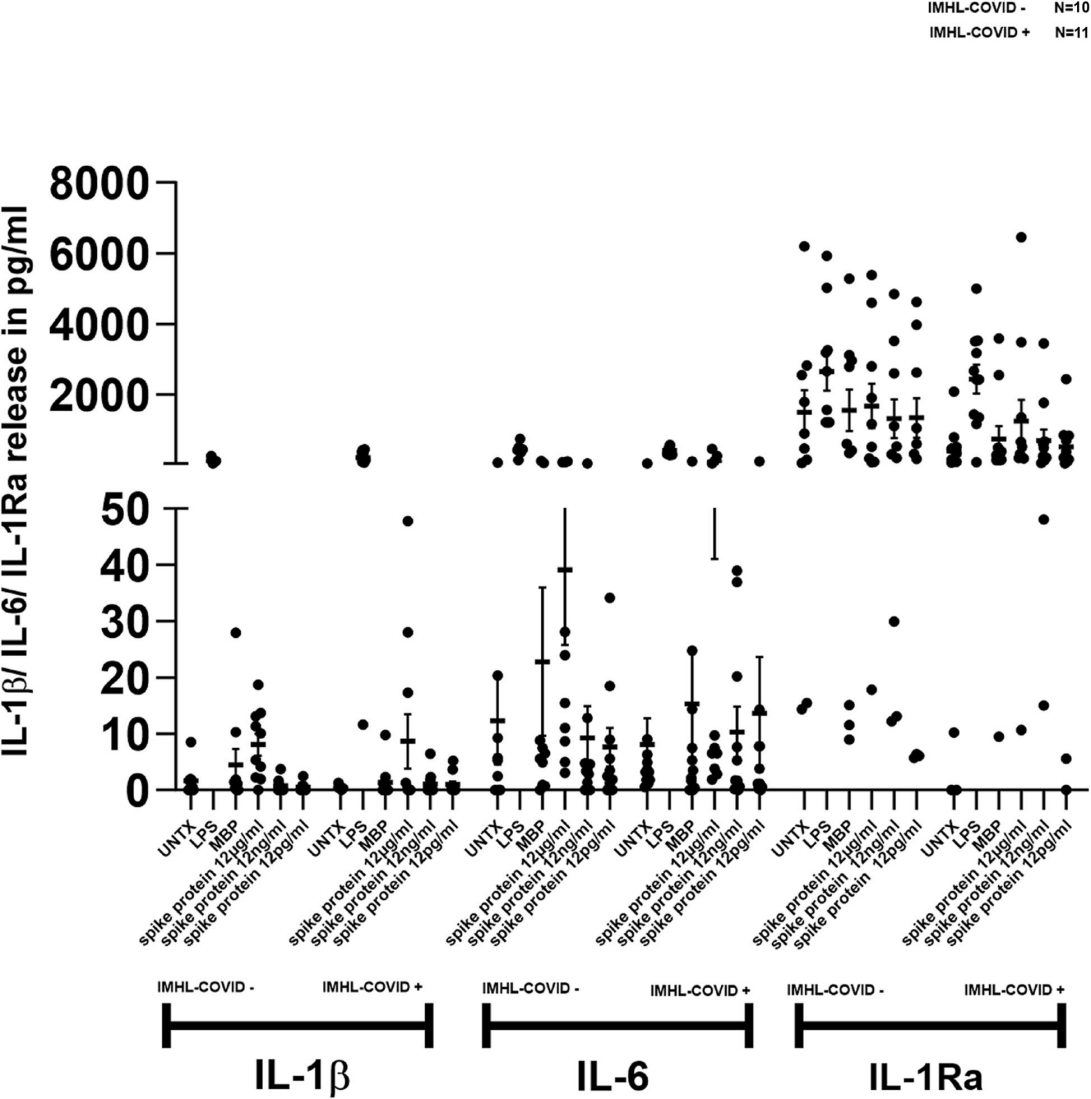
SARS-CoV-2 spike protein treatment resulted in IL-1β and IL-6 release in the PBMCs of IMHL-COVID (+) group. PBMCs from IMHL-COVID (-) patients (*n* = 10) and IMHL-COVID (+) patients (*n* = 11) were treated with spike proteins at the concentrations of 12 μg/ml, 12 ng/ml, 12 pg/ml, MBP as nonspecific protein, LPS as positive control and compared with no treatment. Levels of IL-1β/IL-6/IL-1Ra in the conditioned supernatant were determined by sandwich ELISA. IL-1β—SARS-CoV-2 spike protein treatment resulted in IL-1β release in both groups with slightly higher in IMHL-COVID (+) group. IL-6—SARS-CoV-2 spike protein induces greater levels of IL-6 release in IMHL-COVID (+) patients compared to IMHL-COVID (-) patients and was more robust in IMHL-COVID (+) patients compared to IMHL-COVID (-) patients. IL-1Ra—SARS-CoV-2 spike protein induces IL-1Ra release in the PBMCs of both groups in a dose dependent manner. IMHL-COVID (-) patients showed slightly higher levels compared to IMHL-COVID (+) patients

IL-1β, IL-6 and IL-1RA mRNA expression is mildly enhanced in response to spike protein in IMHL-COVID (+) patients as compared to their IMHL-COVID (-) counterpart. (Fig. 1). Given the mRNA induction of IL-1β, IL-6 and IL-1Ra after spike protein induction we asked if similar pattern is followed at released protein level as well. SARS-CoV-2 spike protein at 12 μg/ml concentration induces robust release of IL-6 in IMHL-COVID (+) patients compared to IMHL-COVID (-) patients (Fig. 2). SARS-CoV-2 spike protein treatment resulted in IL-1β release in both groups (Fig. 2). SARS-CoV-2 spike protein at 12 μg/ml concentration resulted in an increased release of IL-1Ra in the PBMCs of both groups in a dose dependent manner. IMHL-COVID (-) patients showed slightly greater release of IL-1Ra when compared to IMHL-COVID (+) patients (Fig. 2), suggesting an anti-inflammatory effect.

### IL-6 cytokine expression and release is predominantly from COVID ( +) AIED patients

We segregated IMHL-COVID (+) patients into two groups, AIED (*n* = 6) and SSNHL (*n* = 5), based on whether the patient had experienced previous hearing declines unrelated to COVID-19 and compared the results of both mRNA expression and cytokine release from PBMCs.

**Fig. 3:**
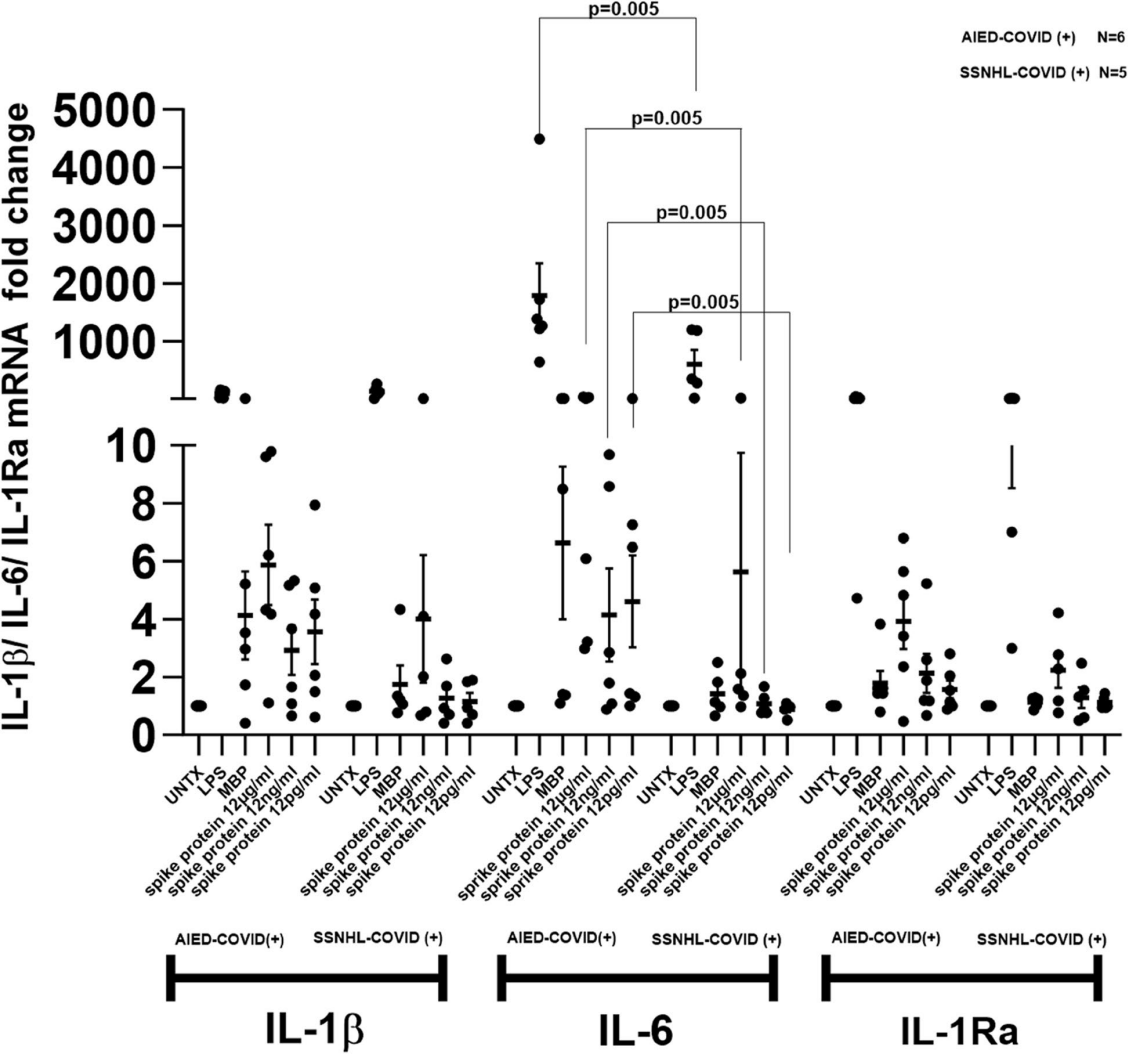
IMHL-COVID (+) patients (*n* = 11) patients were segregated into two groups AIED-COVID (+) (*n* = 6) and SSNHL-COVID (+) (*n* = 5) based on previous hearing decline unrelated to COVID-19. IL-1β—SARS-CoV-2 spike protein at 12 μg/ml concentration showed mild induction IL-1β mRNA expression in both groups of IMHL-COVID (+) patients with slightly higher levels in AIED-COVID (+).IL-6—SARS-CoV-2 spike protein at 12 μg/ml concentration showed robust induction of IL-6 mRNA expression in AIED-COVID (+) group (*n* = 6) when compared with SSNHL-COVID (+) patient group (*n* = 5). Wilcoxon rank sum test with significant *P* values (< 0.005) shown. IL-1Ra—SARS-CoV-2 spike protein at 12 μg/ml concentration showed mild induction IL-1Ra mRNA expression in both groups of COVID (+) IMHL patients. AIED-COVID (+) group (*n* = 6) showed almost twofold higher induction with spike protein at 12 μg/ml when compared with SSNHL-COVID (+) group (*n* = 5)

SARS-CoV-2 spike protein treatment resulted in mild increase in IL-1β mRNA levels in the PBMCs of both groups with slightly higher in IMHL-COVID (+) group (Fig. 3). SARS CoV2 spike protein showed robust induction of IL-6 mRNA expression in AIED-COVID (+) group compared to the SSNHL-COVID (+) group. Wilcoxon's Rank Sum was used to compare between the two groups indicated a significant effect (*p* < 0.005) for the comparison between LPS, 12 μg/ml, 12 ng/ml and 12 pg/ml SARS-CoV-2 spike protein treatments in both groups (Fig. 3). SARS-CoV-2 spike protein at 12 μg/ml concentration showed almost twofold higher induction of IL-1Ra mRNA expression in AIED-COVID (+) group (*n* = 6) when compared with SSNHL-COVID (+) group (*n* = 5) (Fig. 3).

**Fig. 4:**
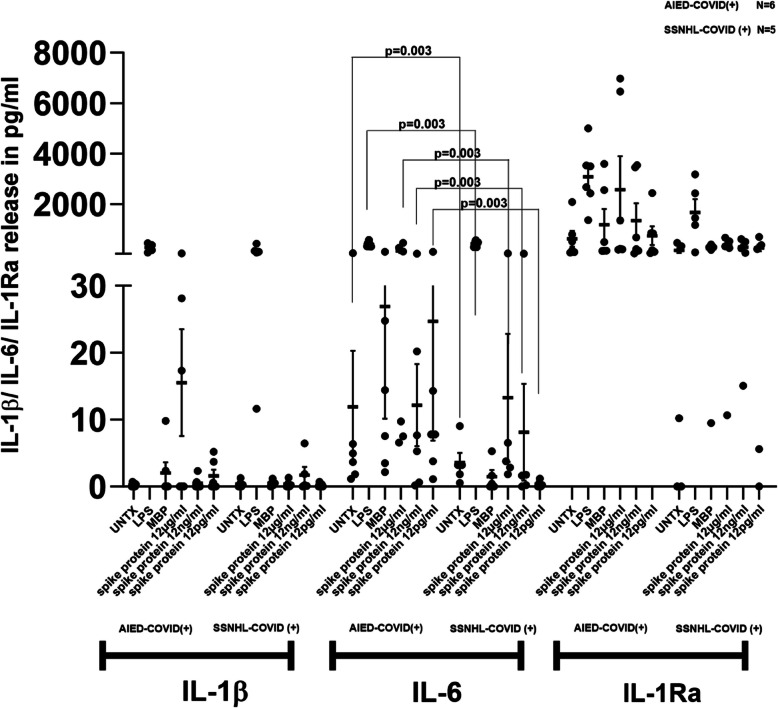
IMHL-COVID (+) patients (*n* = 11) patients were segregated into two groups AIED-COVID (+) and SSNHL-COVID (+) based on previous hearing decline unrelated to COVID-19. IL-1β—SARS-CoV-2 spike protein treatment resulted in moderate increase in IL-1β release in AIED-COVID (+) group whereas almost no induction in SSNHL-COVID (+) group. IL-6—SARS CoV2 spike protein showed robust induction of IL-6 release in AIED-COVID (+) group compared to the SSNHL-COVID (+) group. Wilcoxon's Rank Sum was used to compare between the two groups indicated a significant effect (*p* < 0.003) for the comparison between untreated, LPS, 12 μg/ml, 12 ng/ml and 12 pg/ml treatments in both groups. IL-1Ra- SARS-CoV-2 spike protein at 12 μg/ml concentration showed mild induction IL-1Ra mRNA expression in both groups of COVID (+) IMHL patients. AIED-COVID (+) group (*n* = 6) showed almost sevenfold higher induction with spike protein at 12 μg/ml when compared with SSNHL-COVID (+) group (*n* = 5)

SARS-CoV-2 spike protein treatment resulted in moderate increase in IL-1β release in AIED-COVID (+) group whereas almost no induction in SSNHL-COVID (+) group (Fig. 4). SARS CoV2 spike protein showed robust induction of IL-6 release in AIED-COVID (+) group compared to the SSNHL-COVID (+) group. Wilcoxon's Rank Sum was used to compare between the two groups indicated a significant effect (*p* < 0.003) for the comparison between untreated, LPS, 12 μg/ml, 12 ng/ml and 12 pg/ml SARS-CoV-2 spike protein treatments in both groups (Fig. 4). SARS-CoV-2 spike protein at 12 μg/ml concentration showed almost sevenfold higher induction of IL-1Ra mRNA expression in AIED-COVID (+) group (*n* = 6) when compared with SSNHL-COVID (+) group (*n* = 5) and sustained release at the lower concentrations of spike protein. (Fig. 4).

### COVID infection results in greater pro-inflammatory cytokine expression than vaccination

IMHL-COVID (+) patients (*n* = 11) patients were segregated into two groups, vaccination (*n* = 7) and infection (*n* = 4), based on their hearing loss status due to COVID-19 vaccine administration or COVID-19 infection. The infection group was comprised of 4 patients, 3 AIED and 1 SSNHL. The vaccination group was comprised of 3 AIED and 4 SSNHL.

**Fig. 5:**
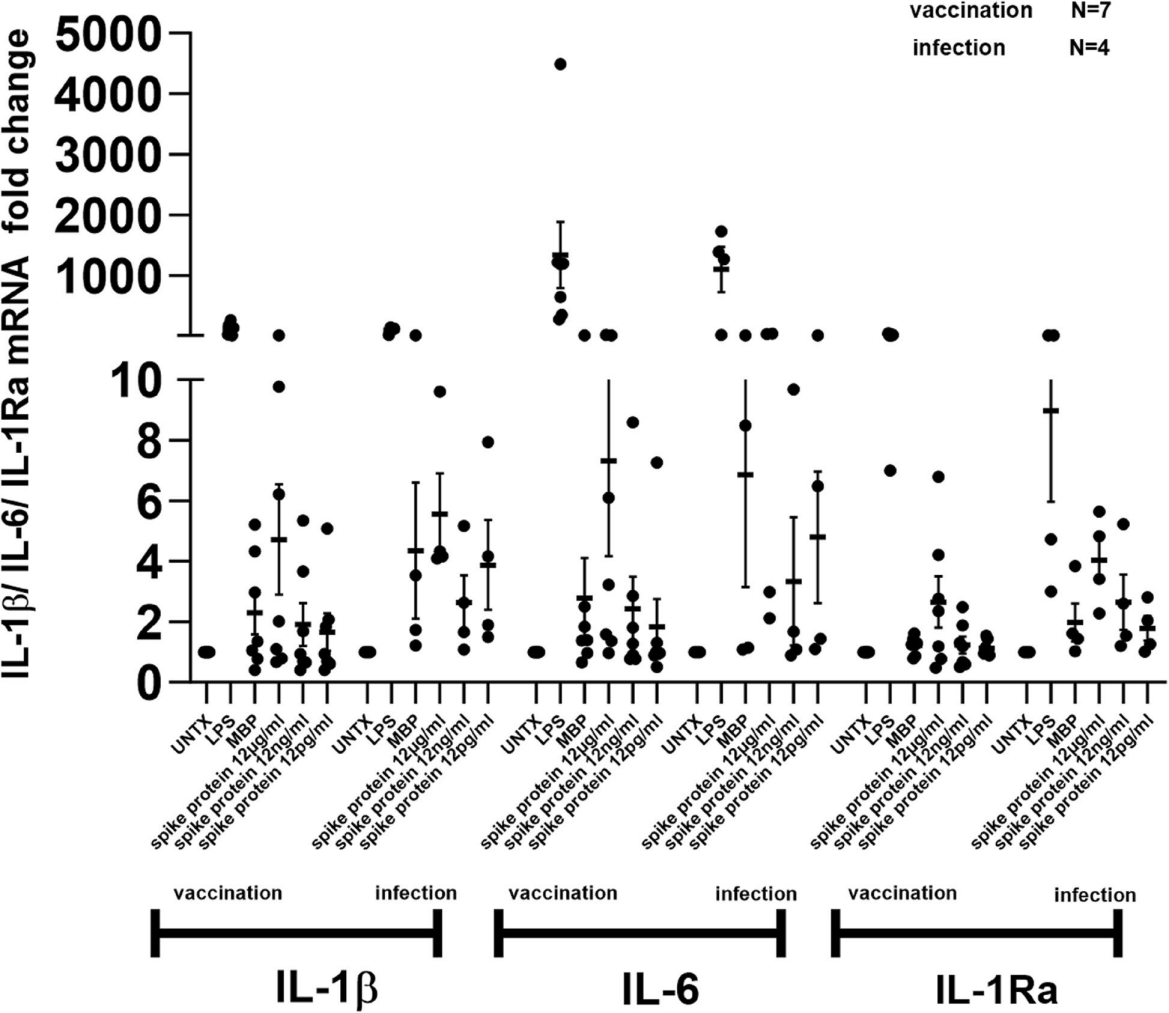
IMHL-COVID (+) patients (*n* = 11) patients were segregated into two groups (vaccination (*n* = 7) and infection (*n* = 4)) based on their hearing loss status due to COVID-19 vaccine administration or COVID-19 infection. IL-1β**—**SARS-CoV-2 spike protein at 12 μg/ml concentration showed mild induction of IL-1β mRNA expression in both groups of IMHL-COVID (+) patients with slightly higher levels in the infection group compared to vaccination group. IL-6—SARS-CoV-2 spike protein at 12 μg/ml concentration showed robust induction of IL-6 mRNA expression in infection group (*n* = 4), when compared with the vaccination group (*n* = 7). IL-1Ra**-**SARS-CoV-2 spike protein at 12 μg/ml concentration showed mild induction in both groups

**Fig. 6:**
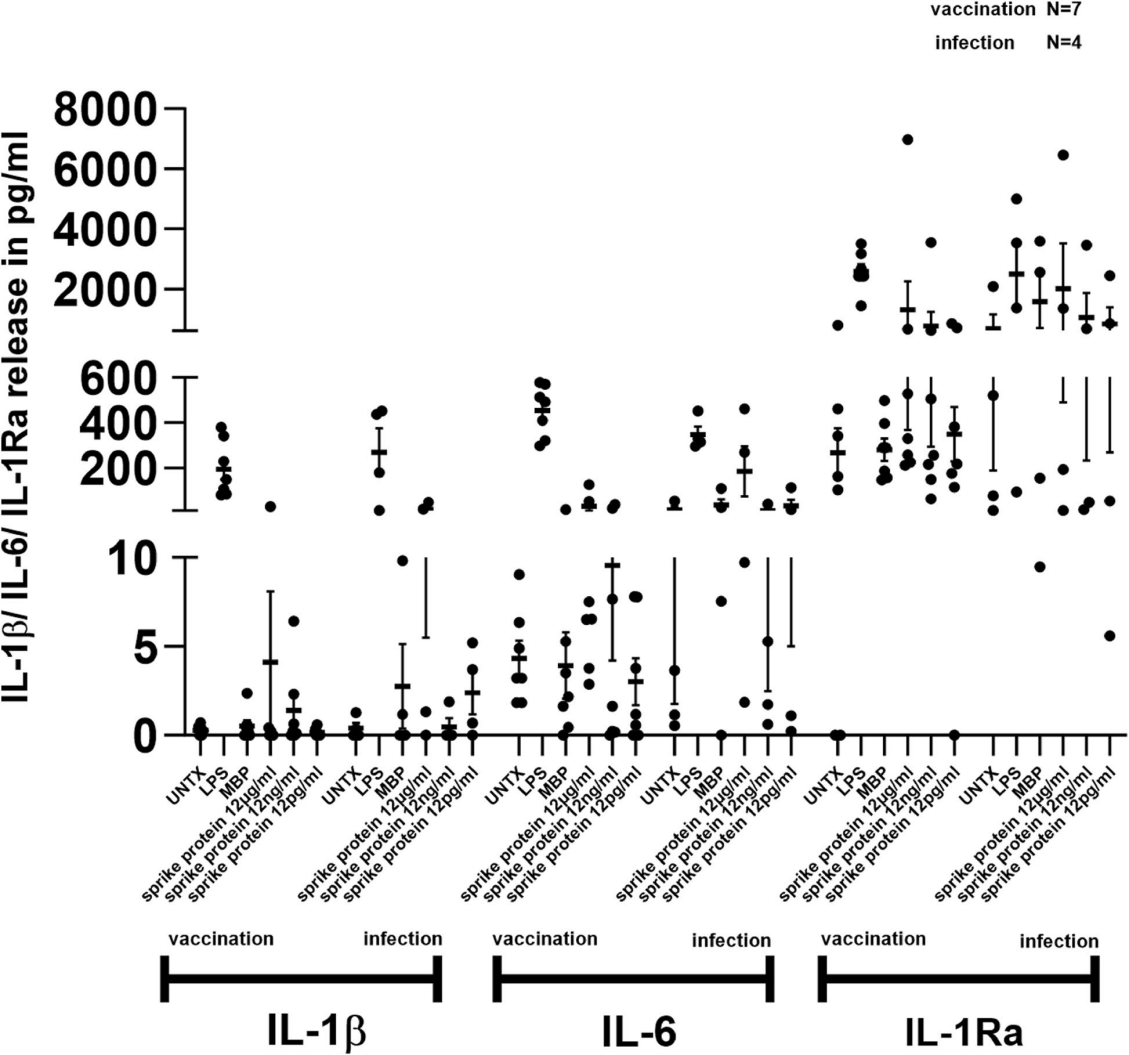
IMHL-COVID (+) patients (*n* = 11) patients were segregated into two groups (vaccination (*n* = 7) and infection (*n* = 4)) based on their hearing loss status due to COVID-19 vaccine administration or COVID-19 infection. IL-1β**—**SARS-CoV-2 spike protein at 12 μg/ml concentration showed strong induction of IL-1β release in infection group (*n* = 4) group, more than fourfold when compared to vaccination group (*n* = 7). IL-6—SARS-CoV-2 spike protein at 12 μg/ml concentration showed robust release of IL-6 in infection group (*n* = 4), more than sixfold when compared to vaccination group (*n* = 7). IL-1Ra- SARS-CoV-2 spike protein showed dose dependent release of IL-1Ra in both groups. Spike protein at 12 μg/ml concentration showed robust release of IL-1Ra in infection group (*n* = 4) when compared to vaccination group (*n* = 7)

SARS-CoV-2 spike protein at 12 μg/ml concentration showed mild induction of IL-1β mRNA expression with slightly higher levels in infection group compared to vaccination group (Fig. 5). SARS-CoV-2 spike protein at 12 μg/ml concentration showed robust induction of IL-6 mRNA expression in infection group when compared with vaccination group. For IL-1Ra showed mild induction of mRNA expression in both groups (Fig. 5). SARS-CoV-2 spike protein at 12 μg/ml concentration showed strong induction of IL-1β release in infection group (around fourfold) when compared with vaccination group (Fig. 6). SARS-CoV-2 spike protein at 12 μg/ml concentration showed robust release of IL-6 in infection group (around sixfold) when compared to the vaccination group. SARS-CoV-2 spike protein showed dose-dependent release of IL-1Ra in both groups. Spike protein at 12 μg/ml concentration showed much greater release of IL-1Ra in infection group when compared to the vaccination group (Fig. 6).

## Discussion

In the present study, we attempted to establish an association between COVID-19 exposure and pro-inflammatory cytokine expression in PBMC from IMHL patients reporting an acute decline in hearing after COVID-19 infection or vaccination. Notably, all AIED patients that experienced a hearing decline in absence of COVID exposure were uneventfully vaccinated prior to study inclusion. Because we have previously observed dysregulation of IL-1β Pathak et al. 2011 and IL-6 Gorthey et al. 2021 in patients with corticosteroid-resistant AIED, we queried whether these cytokines similarly were involved in the perceived hearing decline in patients exposed to COVID-19 through infection or vaccination, and whether corticosteroid-response influenced cytokine expression. Both AIED and Meniere’s patients exhibit immune hyper-reactivity, with periods of unexplained hearing fluctuation, with or without vertigo in response to unclear environmental stimuli Wichova et al. 2021. We observed IL-1β and IL-6 induction in response to spike protein was much stronger in the patients with IMHL after COVID-19 infection or vaccination (COVID +) compared to IMHL that experienced a hearing decline unrelated to COVID exposure (COVID-), despite the fact these IMHL patients had been previously vaccinated. Furthermore, when this group was segregated, the AIED-COVID (+) subgroup was responsible for the observed differences. The AIED-COVID (+) group mounted a robust induction of IL-1β, and IL-6 release from PBMC in response to SARS CoV2 spike protein when compared to the SSNHL-COVID (+) group (Fig.4). This induction appeared irrespective of clinical corticosteroid response, however it is difficult to assert with certainty, as the number of subjects is likely too small to draw meaningful conclusions. We hypothesize that the AIED-COVID (+) group which had hearing declines prior to COVID-19 exposure or vaccination already had an aberrant hyperactive immune system which was augmented with COVID-19 exposure. These observations would suggest that some AIED patients may be more predisposed to COVID-mediated inflammation.

The mechanism of immune hyper-reactivity of AIED patients is largely unknown, and why some autoimmune patients can contract COVID-19 infection without hearing sequelae or undergo uneventful vaccination and others appear to mount pro-inflammatory responses is unclear. Anti-cochlin antibodies have been demonstrated in AIED patients Baek 2006. We have also previously reported that cochlin shares sequence homology with some mold species, providing a potential mechanism for environmental exposure triggering sensorineural hearing loss in these patients Pathak et al. 2013. An amino acid sequence alignment was performed comparing SARS CoV-2 spike protein with the inner ear protein cochlin to determine whether immune-cross-reactivity could be a mechanism for developing SNHL. No protein sequence homology between cochlin and the SARS CoV-2 spike protein could be identified (not shown).

Although we identified a subgroup of patients that clinically reported an acute hearing decline and observed biologic, pro-inflammatory responses in their PBMC, there are limitations in our study. First, we measured immune responses to purified spike protein rather than response to COVID-19 vaccines or live virus. Both vaccines, due to their adjuvants and live virus, may mount stronger immune responses, however the amount of purified protein used may not be physiologic in all cases. In support of the concentration used, the concentrations of spike protein used in this study are of similar range to what George et al*.*observed in the plasma of some COVID-19 patients (0–17.5 µg/mL) George 2021. Second, and perhaps most significant, there was a variable interval from the index event (infection or vaccination) and the time the patient’s office visit where the hearing loss was confirmed and PBMC were harvested for study, despite the enrollment criteria of a perceived hearing change within 2 weeks of the sentinel event. Although responses to spike protein likely involve memory T cell responses, and therefore potentially may make the elapsed time less significant, larger cytokine responses may have been observed if PBMC were harvested immediately post sentinel event.

Interestingly, when we segregated infection from vaccination, the COVID positive infection group mounted even stronger responses than the vaccinated group, again largely driven by the AIED patients within this group. Furthermore, the data did not support an increased risk to patients with SSNHL, as no significant inflammation was observed to spike protein in this cohort. Patients with AIED often mount stronger responses to external stimuli than patients with isolated SSNHL. Future studies will compare covid related hearing loss with the induction of hearing loss to other seasonal viruses such as influenza, to determine if this immunologic hyperreactivity is indeed specific.

## Conclusions

In summary, our observations suggest that some AIED patients are at risk of COVID-mediated hearing loss following COVID-19 infection. Given the comparator group of COVID (-) patients were AIED patients, it suggests not all AIED patients were at risk, as this group were uneventfully vaccinated for study inclusion. Factors that confer increased risk in the COVID (+) AIED subgroup remain unknown. IL-1β and IL-6 mRNA expression and release in response to spike protein was higher in IMHL-COVID (+) group than their IMHL-COVID (-) counterparts during an acute decline in hearing, Although AIED (+) group transcribed and released more IL-1β and IL-6 when compared to SSNHL group, AIED-COVID (+) infection group released greater amounts of IL-1β and IL-6 into the culture supernatants as compared with the vaccination group. Additionally, a cohort of AIED patients mounted no significant inflammation to spike protein and were uneventfully vaccinated (IMHL-COVID (–)). Results obtained and data interpretation should be tempered by the small sample size and the variable period from sentinel event to PBMC acquisition. Based on these preliminary observations, vaccination should be undertaken in all AIED patients, however caution should be exercised in undergoing vaccination during periods of acute disease activity for two reasons. First, during periods of active hearing fluctuation, the antigenic exposure may trigger an immune response and instigate further hearing loss in some AIED patients. Second, the temporal relationship between vaccination and hearing loss may lead the involved patient and practitioner to erroneously conclude the two events are related. In support of this second conclusion, we failed to observe any significant IL-1β mediated inflammation in the COVID -SSNHL group suggesting either non-IL-1β -mediated inflammation or, more likely, the patients experienced a SSNHL that is unrelated to COVID-19.

## Data Availability

All data generated or analyzed during this study are included in this article. The datasets from the current study are available from the corresponding author on reasonable request.

## Abbreviations

ACE2: Angiotensin-converting enzyme 2
AIED: Autoimmune inner ear disease
CARD: Caspase-associated recruitment domain
COVID-19: Coronavirus disease
FURIN: FES upstream region
IMHL: Immune-mediated hearing loss
IL: Interleukin
IL-1Ra: IL-1 receptor antagonist
IRB: Institutional Review Board
kDa: Kilo Daltons
MD: Meniere's disease
MBP: Myelin basic protein
MS: Multiple sclerosis
NLRP3: Nucleotide-binding domain, leucine-rich–containing family, pyrin domain–containing-3
PBMC: Peripheral blood mononuclear cell
SSNHL: Sudden sensorineural hearing loss
SARS-CoV-2: Severe acute respiratory syndrome coronavirus 2
TMPRSS: Transmembrane protease serine
UNTX: Untreated
